# Risk factors for the recurrence of relapsing polychondritis

**DOI:** 10.1186/s13075-022-02810-0

**Published:** 2022-05-30

**Authors:** Tsuneyasu Yoshida, Hajime Yoshifuji, Mirei Shirakashi, Akiyoshi Nakakura, Kosaku Murakami, Koji Kitagori, Shuji Akizuki, Ran Nakashima, Koichiro Ohmura, Akio Morinobu

**Affiliations:** 1 Department of Rheumatology and Clinical Immunology, Graduate School of Medicine, Kyoto University, Kyoto, Japan; 2 Department of Biomedical Statistics and Bioinformatics, Kyoto University Graduate School of Medicine, Kyoto, Japan

**Keywords:** Relapsing polychondritis, Recurrence, Glucocorticoid, Immunosuppressant, Biologics

## Abstract

**Background:**

Although the survival rates of patients with relapsing polychondritis (RP) have increased remarkably, the high recurrence rate remains a significant concern for physicians and patients. This retrospective study aimed to investigate the risk factors for RP recurrence.

**Methods:**

Patients with RP who presented to Kyoto University Hospital from January 2000 to March 2020 and fulfilled Damiani’s classification criteria were included. Patients were classified into recurrence and non-recurrence groups. Risk factors for RP recurrence were analysed using a Cox proportional hazards model, and Kaplan–Meier survival curves were drawn.

**Results:**

Thirty-four patients were included. Twenty-five patients (74%) experienced 64 recurrences (mean: 2.56 recurrences per patient). The median duration before the first recurrence was 202 [55−382] days. The median prednisolone dose at the initial recurrence was 10 [5−12.75] mg/day. Tracheal involvement was significantly more frequent in the recurrence group at the initial presentation (44.0% vs. 0.0%, *p*=0.0172) than in the non-recurrence group, and pre-treatment C-reactive protein levels were significantly higher in the recurrence group than in the non-recurrence group (4.7 vs 1.15 mg/dL, *p*=0.0024). The Cox proportional hazards model analysis revealed that tracheal involvement (hazard ratio [HR] 4.266 [1.535−13.838], *p*=0.0048), pre-treatment C-reactive protein level (HR 1.166 [1.040−1.308], *p*=0.0085), and initial prednisolone monotherapy (HR 4.443 [1.515−16.267], *p*=0.0056) may be associated with recurrence. The median time before the initial recurrence was significantly longer in patients who received combination therapy with prednisolone and immunosuppressants or biologics (400 vs. 70 days, *p*=0.0015).

**Conclusions:**

Tracheal involvement, pre-treatment C-reactive protein level, and initial prednisolone monotherapy were risk factors for recurrence in patients with RP. Initial combination therapy with prednisolone and immunosuppressants may delay recurrence.

## Background

Relapsing polychondritis is a rare disease that causes inflammation of the chondrocytes of the ear, nose, and trachea, and it can affect any organ of the body [1]. Although corticosteroid therapy is the mainstream treatment for relapsing polychondritis (RP), immunosuppressive drugs, such as conventional synthetic disease-modifying anti-rheumatic drugs [2, 3], biologics [4], and JAK inhibitors [5], have been used empirically in cases of treatment resistance or severe cases. The 5-year and 10-year survival rates of RP were 74% and 55%, respectively, in 1986, whereas higher rates (95% and 91%, respectively) were reported in 2016 [6, 7]. This improvement is considered to be due to the early detection of RP, the availability of new immunosuppressive drugs, and intensified treatment by individual physicians [7].

However, achieving remission for RP remains challenging. A French study showed that only 19% of the patients achieved complete treatment response in the first 6 months, despite the use of biologics [4]. Furthermore, a recent study showed that most patients with RP have persistent disease activity, despite treatment [8]. In addition, the recurrence of symptoms that require intensive treatment is often encountered in clinical practice and poses a considerable concern for physicians and patients. Repeat recurrences of RP can lead to irreversible organ damage. Particularly, in patients with tracheal chondritis, chronic repetitive inflammation can cause irreversible destruction and fibrosis of the trachea, resulting in a high risk of mortality [9, 10]. Therefore, it is crucial to understand the risk factors for RP recurrence, for preventive purposes. However, owing to the rarity of this disease, few reports are available in the literature. Thus, the present study aimed to retrospectively analyse the records of 34 patients with RP to determine the risk factors associated with RP recurrence.

## Methods

### Patients

Patients who presented with RP at Kyoto University Hospital from January 2000 to March 2020 and fulfilled Damiani's classification criteria [11] were included in this study. Patients who were followed up for < 1 year or those who had a history of glucocorticoid use for the treatment of other diseases (i.e. concomitant autoimmune diseases, such as systemic lupus erythematosus, mixed connective tissue disease, and dermatomyositis) before RP treatment were excluded. Auricular, nasal, and tracheal cartilage involvement were diagnosed based on physical examination, imaging (computed tomography and positron emission tomography-computed tomography), or pathohistological findings. RP relapse was defined as (1) worsening or newly developed symptoms related to RP, which were detected by physical examination or imaging studies and had led to the intensification of treatment or (2) the elevation of the levels of inflammatory biomarkers (C-reactive protein [CRP] and erythrocyte sedimentation rate [ESR]) from basal levels, which was considered to be due to the activity of RP and had led to the intensification of treatment.

### Evaluation of clinical laboratory parameters

Blood tests included tests for CRP level (normal range ≤0.3 mg/dL), ESR (≤10/h), ferritin level (<280 ng/mL), IgG level (820−1740 mg/dL), and white blood cell count, which were measured by standard methods. Anti-type II collagen antibody level was measured via the enzyme-linked immunosorbent assay technique (Mayo Clinic Laboratories, cut-off > 25 EU/mL).

### Statistical analysis

The chi-square test and Wilcoxon’s rank-sum test were used to analyse categorical and numerical variables, respectively. Survival curves for treatment were drawn via the Kaplan–Meier method. Univariate analysis was performed to explore the risk factors for RP relapse. Hazard ratios (HRs) and 95% confidence intervals (CIs) were estimated using the Cox proportional hazards model. If no relapse was observed for more than a year, the data were time-censored in the first year. Statistical analyses were performed using JMP version 14 and SAS version 9.4 (SAS Institute Inc., Cary, NC, USA). A *p* value <0.05 was considered significant.

## Results

### Patient characteristics

**Fig. 1:**
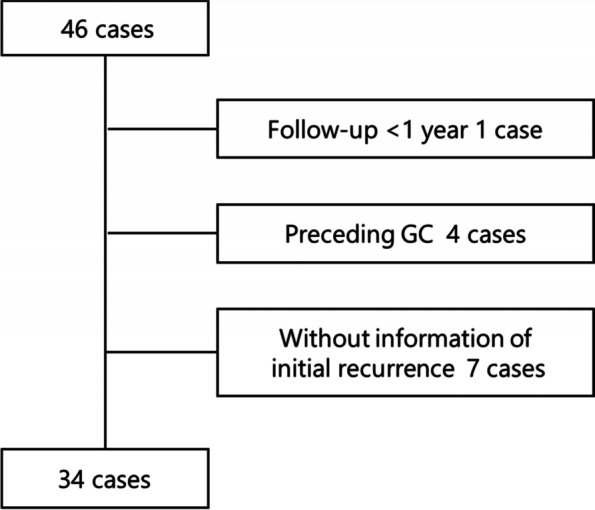
Flow chart of patient selection. Forty-six patients with RP were selected from medical records. We excluded 4 patients who were followed up for < 1 year, 1 patient who previously received glucocorticoids for the treatment of other diseases, and 7 patients with missing data. Finally, a univariate analysis was performed with the data of 34 patients

Thirty-four patients with RP were selected after exclusions (Fig. 1). The clinical characteristics of the 34 patients are shown in Table 1 (left column). There were 17 female patients (50.0%), the median age was 49 (40−67) years, and the median disease duration was 5.0 [2.5−6.8] years. Two patients (5.9%) had Behçet’s disease and two other patients (5.9%) had malignancies (gastric cancer and myelodysplastic syndromes). The most common initial symptom was auricular chondritis (67.6%), followed by tracheal chondritis (32.4%) and arthritis (29.4%).

**Table 1:** Patient characteristics

	Total	Patients with recurrence	Patients without recurrence	***P*** value
Patients, *n*	34	25	9	
Female, *n* (%)	17 (50.0)	12 (48.0)	5 (55.6)	1.0000
Median age at onset, years	49 [40-67]	51 [39-66]	65 [38-74]	0.3587
Disease duration, years	5.0 [2.5-6.8]	5.0 [2.0-6.9]	4.5 [2.7-6.7]	0.6819
Time until diagnosis, days	150 [86-265]	151 [83-236]	149 [77-306]	0.9223
Time until treatment, days	153 [84-270]	151 [82-245]	175 [81-378]	0.4944
Autoimmune disease, *n* (%)	3 (8.8)	3 (12.0)	0 (0.0)	0.5488
Behçet’s syndrome	2 (5.9)	2 (100.0)	0 (0.0)	
Malignancy, *n* (%)	2 (5.9)	2 (8.0)	0 (0.0)	1.0000
Myelodysplastic syndromes	1 (2.9)	1 (100.0)	0 (0.0)	
Initial symptoms, *n* (%)				
Median number of initial symptoms, *n*	1 [1-2]	1 [1-2.5]	1 [1-2]	0.385
Auricular involvement	23 (67.6)	15 (60.0)	8 (88.89)	0.2137
Tracheobronchial involvement	11 (32.4)	11 (44.0)	0 (0.0)	0.0172
Articular involvement	10 (29.4)	9 (36.0)	1 (11.1)	0.2250
Nasal involvement	5 (14.7)	3 (12.0)	2 (22.2)	0.5908
Eye involvement	4 (11.8)	4 (16.0)	0 (0.0)	0.5536
Vestibulocochlear involvement	3 (8.8)	2 (8.0)	1 (11.1)	1.0000
Preceding infection	10 (29.4)	8 (32.0)	2 (22.2)	0.6921
Baseline RPDAI	25 [19-35]	30 [19.5-41.5]	23 [13.5-29.5]	0.0786
Baseline laboratory data				
WBC *n*=24^a^ (/μl)	7200 [5870-8188]	7700 [6080-8600], n=15	7000 [5375-8025], n=9	0.6798
Neutrophil *n*=24^a^ (/μl)	4774 [3691-5971]	5605 [3806-5986], n=15	4613 [3156-5745], n=9	0.4561
Lymphocyte *n*=24^a^ (/μl)	1595 [1248-2029]	1519 [1232-2070], n=15	1798 [1301-2102], n=9	0.4929
Monocyte *n*=24^a^ (/μl)	435 [346-542]	468 [297-589], n=15	378 [272-451], n=9	0.0786
Hb *n*=25^a^ (g/dl)	12.3 [10.8-13.8]	11.6 [10.4-13.9], n=16	13.0 [11.8-13.9], n=9	0.1407
Plt *n*=25^a^ (×10^4^/μl)	30.3 [24.5-38.1]	32.5 [24.5-46.5], n=16	30.3 [24.4-30.7], n=9	0.1407
CRP *n*=25^a^ (mg/dl)	3.3 [0.72-6.85]	4.7 [2.60-9.81], n=16	1.15 [0.10-3.10], n=9	0.0024
ESR *n*=22^a^ (mm/h)	70 [27-101.5]	78 [40-107], n=15	28 [16-83], n=7	0.0722
IgG *n*=22^a^ (mg/dl)	1455 [1140-1836]	1704 [1249-1982], n=16	1159 [984-1431], n=6	0.0325
Anti-type II collagen antibody (+) *n*=26^a^, *n* (%)	13 (50.0)	8 (47.1), n=17	5 (55.6), n=9	1.0000
Whole treatment				
Glucocorticoid, *n* (%)	34 (100)	25 (100)	9 (100)	
Initial prednisolone dose, *n*=33^a^ (mg)	30 [15-47.5]	30 [15-48.75]	15 [10-40]	0.2717
Immunosuppressant, *n* (%)	24 (70.6)	23 (92.0)	2 (22.2)	<0.001
Methotrexate	20 (83.3)	18 (72.0)	2 (22.2)	0.0168
Cyclophosphamide	8 (33.3)	8 (32.0)	0 (0.0)	0.0770
Azathioprine	7 (29.2)	7 (28.0)	0 (0.0)	0.1506
Tacrolimus	6 (25.0)	5 (20.0)	1 (11.1)	1.0000
Cyclosporine A	2 (8.3)	2 (8.0)	0 (0.0)	1.0000
Biologics, *n* (%)	15 (44.1)	12 (48.0)	3 (33.3)	0.6974
Tocilizumab	12 (80.0)	10 (40.0)	2 (22.2)	0.4385
Infliximab	7 (46.7)	6 (24.0)	1 (11.1)	0.6445
Adalimumab	1 (6.7)	1 (100.0)	0 (0.0)	
Outcome				
Airway intervention, *n* (%)	6 (17.6)	6 (24.0)	0 (0.0)	0.1622
Death, *n* (%)	2 (5.9)	1 (4.0)	1 (11.1)	0.4652

Numbers are presented as means (SD) or medians [interquartile range: 25–75%]. The chi-square test was used for categorical variables and Wilcoxon’s rank-sum test was used for numerical variables. Airway interventions included intubation, tracheostomy, and non-invasive positive pressure ventilation. ^a^Only available data were analysed

*CRP* C-reactive protein, *ESR* erythrocyte sedimentation rate, *Hb* haemoglobin, *Plt* platelet, *RPDAI* Relapsing Polychondritis Disease Activity Index, *WBC* white blood cell

The median relapsing polychondritis disease activity index (RPDAI) score before treatment was 25 (19−35). The median CRP level and ESR before treatment were 3.3 (0.7−6.9) mg/dL (*n*=25) and 70 (27−102) mm/h (*n*=22), respectively. Anti-type II collagen antibodies were detected in 50% (13/26) of specimens obtained from the patients. Glucocorticoids (prednisolone) were administered to all patients. Immunosuppressive drugs, most commonly, methotrexate (83.3%, 20/24), followed by cyclophosphamide (33.3%, 8/24), azathioprine (29.2%, 7/24), and tacrolimus (25.0%, 6/24), were prescribed to 70.6% (24/34) of all patients. Biologics, most commonly, tocilizumab (80.0%, 12/15), followed by infliximab (46.7%, 7/15), were administered to 44.1% (15/34) of all patients. Six patients (17.6%) required airway interventions, such as intubation, tracheostomy, and non-invasive positive pressure ventilation. Two deaths (5.9%) were recorded. One patient died of cerebral infarction at the age of 81 years, and the other patient died of senility and dementia at the age of 80 years.

### Characteristics of patients who experienced recurrence

Of the 34 patients, 25 (74%) experienced recurrences (64 recurrences; mean: 2.56 recurrences per patient; minimum 1, maximum 9; 0.16 person-year) (Table 2). Eleven recurrences (11/64, 17.2%) were major events, requiring hospitalization. The median age of the patients at the time of initial recurrence was 50 (40−67) years, and the median duration before the first recurrence was 202 (55−382) days. Six of the initial recurrences were major events (6/25, 24%). The median prednisolone dose at the time of initial RP recurrence was 10 (5–12.75) mg/day. The initial symptoms recurred in 68% (17/25) of the cases, and one patient experienced the recurrence of encephalitis.

**Table 2:** Characteristics of the disease recurrences

	Present study
**Total recurrence**	
Total number of patients with recurrence (number)	25
Total number of recurrences (times)	64
Mean number of recurrences (times/person)	2.56
Person-years	0.16
Major recurrence, *n* (%)	11 (17.2)
Minor recurrence, *n* (%)	53 (82.8)
**Initial recurrence,** ***n*=25**	
Median age at initial recurrence (years)	51 (40–67)
Median days to initial recurrence (days)	202 (55–382)
Major recurrence, *n* (%)	6 (24.0)
Minor recurrence, *n* (%)	19 (76.0)
Median PSL doses at initial recurrence (mg)	10 (5–12.75)
Symptoms at initial recurrence	
Tracheobronchial involvement	10 (40.0)
Auricular involvement	6 (24.0)
Nasal involvement	3 (12.0)
Articular involvement	3 (12.0)
Eye involvement	2 (8.0)
Vestibulocochlear involvement	2 (8.0)
Encephalitis	1 (4.0)
Concordance of initial symptoms and symptoms at initial recurrence	17 (68.0)

Numbers are presented as means (SD) or medians [interquartile range: 25–75%]. Some patients had several symptoms at the initial recurrence. Major recurrence: required admission. Minor recurrence: did not require admission. *PSL* prednisolone

### Risk factors for recurrence

To investigate the risk factors for recurrence, we divided the entire patient cohort (*n*= 34) into recurrence and non-recurrence groups. There were no significant differences in age, sex, disease duration, or observation period between the two groups before treatment (Table 1, middle and right columns). Although there was no significant difference in the number of symptoms at the initial presentation, occurrence of tracheal lesions at the initial presentation was associated with RP recurrence (44.0% vs. 0%, *p*=0.0172). Pre-treatment RPDAI tended to be higher in the recurrence group than in the non-recurrence group (median 30 vs. 23, *p*=0.0786). Serum IgG levels were also significantly higher in the recurrence group than in the non-recurrence group (median: 1704 vs. 1159 mg/dL, *p*=0.0325), although serum IgG levels remained within the normal range. Monocyte count tended to be higher in the recurrence group than in the non-recurrence group (median 468 vs. 378 /μL, *p*=0.0786). Positive results of the anti-type II collagen antibody test were not associated with RP recurrence (47.1% vs. 55.6%, *p*=1.0000). There were no significant differences in the initial prednisolone dose between the recurrence and non-recurrence groups (30 vs. 15 mg, *p*=0.2717). However, immunosuppressive agents (particularly methotrexate) were more commonly used in the recurrence group than in the non-recurrence group (72.2% vs. 22.2%, *p*=0.0168). Regarding prognosis, interventions for airway lesions, such as intubation, tracheostomy, and non-invasive positive pressure ventilation use (six patients), were recorded in the recurrence group alone, although no significant between-group difference was observed (24.0% vs. 0.0%, *p*=0.1622).

We performed a univariate analysis of major risk factors for relapse (Table 3). Pre-treatment CRP level (HR 1.166, 95% CI, 1.040−1.308, *p*=0.0085) was found to be a potential risk factor for RP recurrence. Monocyte count (HR 1.04, 95% CI, 1.000−1.008, *p*=0.0690) also tended to be a risk factor, although no significant between-group difference was observed. The hazard ratio for tracheal lesions was 2.666 (95% CI, 1.014−8.283, *p*=0.0466). After adjusting for the initial treatment, the hazard ratio raised to 4.266 (95% CI 1.535−13.838, *p*=0.0048).

**Table 3:** Univariate analysis of risk factors for recurrence

Dependent variables	Recurrence risk	
Variables	Univariate models HR (CI)	*P* value
Age at diagnosis	1002 (0.978, 1.027)	0.8640
Sex	0.857 (0.348, 2.111)	0.7373
Time until treatment	0.998 (0.995, 1.001)	0.1570
RPDAI	1.010 (0.971, 1.050)	0.6305
Tracheobronchial involvement	2.666 (1.014, 8.283)	0.0466
Adjusted by initial PSL monotherapy	4.266 (1.535, 13.838)	0.0048
Auricular involvement	0.674 (0.264, 1.717)	0.4083
CRP	1.166 (1.040, 1.308)	0.0085
ESR	1.009 (0.994, 1.024)	0.2231
Monocyte	1.004 (1.000, 1.008)	0.0690
IgG	1.000 (0.999, 1.001)	0.3586
Initial PSL monotherapy	2.718 (0.981, 9.565)	0.0547
Adjusted by tracheobronchial involvement	4.443 (1.515, 16.267)	0.0056
Initial PSL dose	1.008 (0.986, 1.030)	0.4802

*CRP* C-reactive protein, *ESR* erythrocyte sedimentation rate, *PSL* prednisolone, *RPDAI* Relapsing Polychondritis Disease Activity Index

### Efficacy of combination therapy with immunosuppressants

To investigate the preventive effect of immunosuppressive drugs on relapse, we divided the entire cohort into two groups: PM group and prednisolone combined with immunosuppressants/biologics (PC) group. Table 4 shows the background of patients in each group. The PC group received the following immunosuppressants and biologics: methotrexate (5 patients), intravenous cyclophosphamide (3 patients), azathioprine (1 patient), infliximab (1 patient), and methotrexate, which was changed to tocilizumab (1 patient). There were no differences in age, sex, disease duration, or observation period between the groups. The rate of concomitant use of immunosuppressants was significantly higher when the initial symptom included tracheal involvement (63.6% vs 17.4%, *p*=0.0160). The dose of prednisolone at the initiation of treatment was not entirely different between the PM and PC groups (28 vs 40 mg, *p*=0.2566). The median prednisolone dose in both groups at relapse was 10 mg, and the rate of prednisolone dose reduction before the first relapse was not significantly different between the two groups (PM 0.1 vs PC 0.03 mg, *p*=0.2691). The median duration before initial RP recurrence was significantly longer in the PC group than in the PM group (400 vs. 70 days, *p*=0.0015).

**Table 4:** Comparison of prednisolone monotherapy (PM) and prednisolone combined with immunosuppressants/biologics (PC) groups

	PM	PC	***P*** value
	***n***=23	***n***=11	
Age at onset (years)	52 (45–70)	46 (30–58)	0.1616
Female, *n* (%)	13 (56.5)	4 (36.4)	0.4646
Disease duration (years)	5.5 (2.9–6.8)	4.5 (1.8–12.9)	0.6720
Time until diagnosis (days)	151 (78–270)	105 (91–235)	0.8684
Time until treatment (days)	151 (78–270)	156 (99–467)	0.6586
Initial symptoms, *n* (%)			
Auricular	18 (78.3)	5 (45.5)	0.1143
Tracheobronchial	4 (17.4)	7 (63.6)	0.0160
RPDAI	23 (15–41)	33 (22–35)	0.2851
WBC (/μl)	7000 (5500–8200), *n*=15	7710 (6325–8375), *n*=9	0.5913
Monocyte (/μl)	444 (308–596), *n*=15	430 (388–471), *n*=9	0.8815
Hb (g/dl)	12.4 (10.8–13.9), *n*=15	11.8 (10.4–13.8), *n*=10	0.6570
Plt (×104/μl)	30.1 (21.5–32.7), *n*=15	33.9 (26.6–49.4), *n*=10	0.0521
CRP (mg/dl)	2.8 (0.5–4.7), *n*=16	4.5 (1.6–8.7), *n*=9	0.4270
ESR (mm/h)	70 (43–104), *n*=12	55 (22–102), *n*=10	0.5977
IgG (mg/dl)	1432 (1229–1709), *n*=12	1597 (984–2020), *n*=10	0.7416
Initial PSL doses (mg)	28 (13.8–41.2), *n*=22	40 (15–70), *n*=11	0.2566
PSL doses at initial recurrence (mg)	10 (5–14), *n*=16	10 (6.5–12), *n*=9	0.9319
PSL dose reduction until initial recurrence (mg/day)	0.1 (0.03–0.4), *n*=15	0.03 (0.015–0.2), *n*=9	0.2691
Time until initial recurrence (days)	70 (26–211)	400 (294–1446)	0.0015

Numbers are presented as means (SD) or medians [interquartile range: 25–75%]. The chi-square test was used for categorical variables and Wilcoxon’s rank-sum test was used for numerical variables

*CRP* C-reactive protein, *ESR* erythrocyte sedimentation rate, *Hb* haemoglobin, *IS* immunosuppressant, *PC* prednisolone combined with immunosuppressants/biologics, *Plt* platelet, *PM* prednisolone monotherapy, *PSL* prednisolone, *RPDAI* Relapsing Polychondritis Disease Activity Index, *WBC* white blood cell

**Fig. 2:**
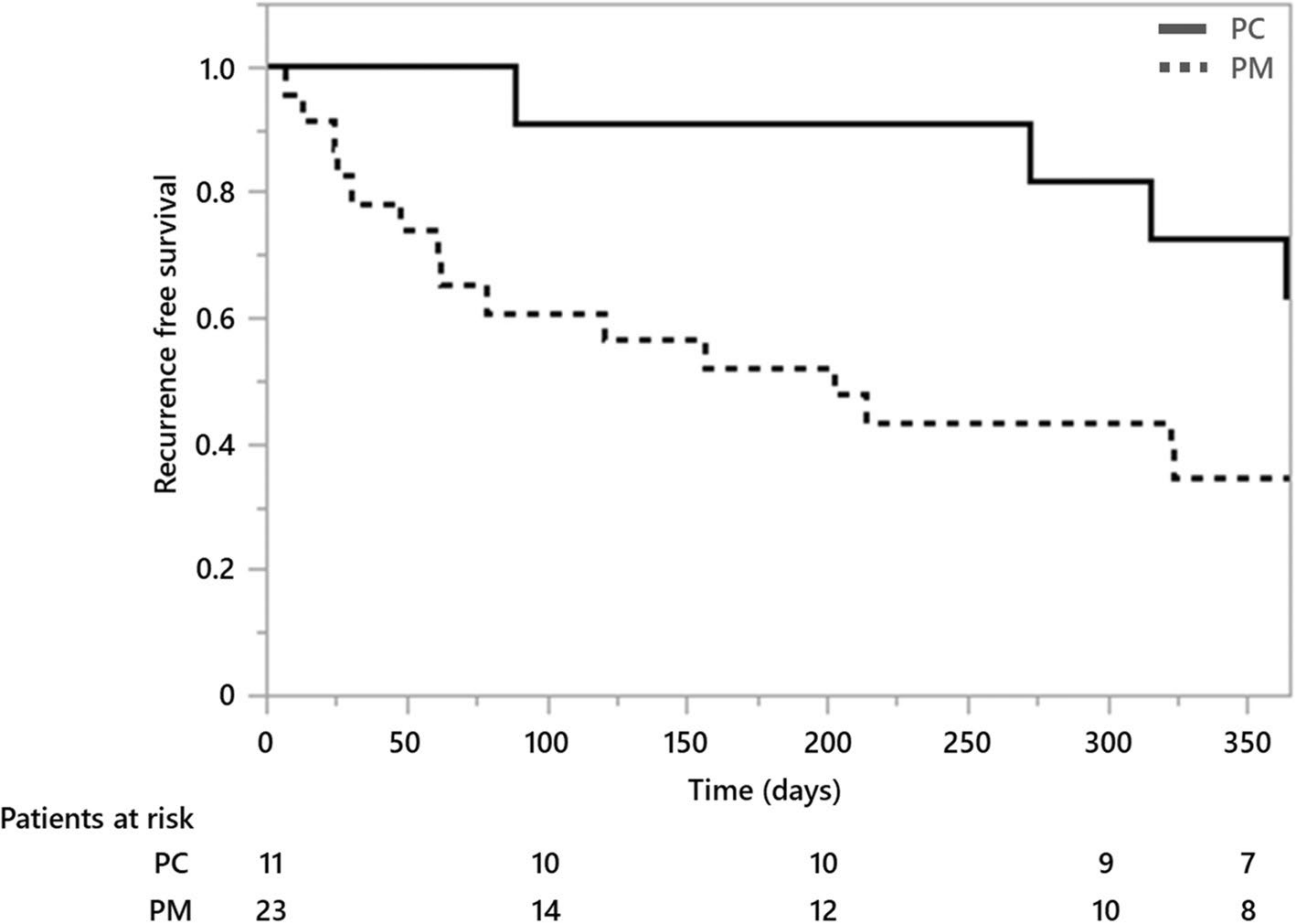
Recurrence-free survival curve of the prednisolone monotherapy (PM) and prednisolone combined with immunosuppressants/biologics (PC) groups. One-year relapse-free survival curves for patients who received prednisolone monotherapy as the initial therapy and for patients who received a combination of prednisolone and immunosuppressants. Wilcoxon’s rank-sum test *p*=0.0372 (log-rank test *p*=0.0655)

Recurrence-free survival curve from the start of treatment is shown in Fig. 2. The hazard ratio for RP recurrence in the PM group versus the PC group was 2.718 (95% CI, 0.981−9.565, *p*=0.0547) (Table 3). After adjusting for the influence of tracheal lesions, the hazard ratio was 4.443 (95% CI 1.515−16.267, *p*=0.0056) (Table 3).

## Discussion

In this study, we retrospectively assessed 34 patients with RP and investigated the risk factors for recurrence, which have not been reported before. The characteristics of the patients (age, sex, initial symptoms, and so on) in our study are essentially similar to those of patients described in previous reports [7, 12]. We observed preceding infection in 29.4% of the total cases. Til et al. reported the cross-antigenicity of cartilage antigen and heat shock protein 60 from *Mycobacterium tuberculosis*, and William et al. reported the association between *Propionibacterium acnes* infection and RP [13, 14]. Based on these reports, it is possible that infection is a trigger of RP.

There were two cases of death during the study period. However, the two cases of death were caused by stroke and senility, which were not considered to be related to RP. In a previous report published in 1986, RP was associated with a 5-year survival rate of 74% and a 10-year survival rate of 55% [6], whereas the corresponding values reported in a recent report published in 2016 were 95% and 91% [7]. In the present study, a similar improvement in survival was observed (median observation period, 4.5 years; survival rate, 95.1%).

The present study highlights a high recurrence rate of RP (74%). A recent study showed that RP had a more persistent active pattern than a relapsing-remitting pattern [8]. However, whether RP assumes a chronic active pattern or a relapsing-remitting pattern may depend on differences in treatment and the rate of therapeutic drug reduction. Moreover, further treatment improvement was considered necessary. We found that the median time before the first relapse was 202 days. The median prednisolone dose at the time of recurrence was 10 mg, consistent with an expert’s opinion, which suggests that a 5−10-mg maintenance dose of prednisolone is suitable for RP [15]. In our analysis, 68% of the patients had similar symptoms at the initial recurrence, while the symptoms in 32% of the patients at the time of relapse were different from the initial symptoms. This suggests that various symptoms of RP should be considered at the time of recurrence. One patient had auricular chondritis as the initial symptom but had encephalitis symptoms at the time of RP recurrence. This finding is consistent with those of Shimizu et al.’s [16] cluster analyses, which showed the relationship between auricular chondritis and encephalitis.

In the analysis of the risk of recurrence, tracheal involvement, pre-treatment CRP level, and PM were identified as potential risk factors for recurrence. Tracheal involvement was associated with death in previous studies [17–19], and the possible mechanisms include ventilation insufficiency or concomitant infection due to airway narrowing or collapse [20, 21]. The findings of the present study suggested that tracheal involvement is a risk factor for recurrence, and that repeat recurrences may lead to irreversible structural changes in the bronchi.

Pre-treatment RPDAI score was significantly higher in the recurrence group than in the non-recurrence group, although pre-treatment RPDAI was not a recurrence risk factor in the univariate analysis. The RPDAI is an index of activity that scores symptoms and examination findings in RP, using a scale of 1–24 points [22]. A relatively high score of 14 or 24 points is assigned to tracheal chondritis, depending on the presence or absence of acute respiratory failure. The recurrence group had a significantly higher median RPDAI score, which may have been influenced by tracheal involvement. A new assessment scale for assessing disease activity is required.

Pre-treatment CRP levels were also identified as risk factors for recurrence in the present study, although the results differ from those of several previous reports. Consistent with our study, which suggests that a high pre-treatment CRP level is a risk factor for recurrence, a case series report indicated that CRP level was elevated at the time of RP recurrence [23]. However, in some other reports, more than 10% of the patients had normal CRP levels at the time of recurrence [9], and CRP level was not related to disease activity [24, 25]. According to these two reports [24, 25], CRP level was measured in patients who were receiving treatment, and the treatment may have affected the level of this inflammatory marker.

Finally, patients who underwent PM were at a higher risk of recurrence than those who received the combination therapy. The risk ratio for recurrence increased when adjustments were made for the presence of initial airway involvement, suggesting the importance of PC therapy, regardless of the involved organ at RP onset.

There were several limitations in the present study. First, it was a single-centre, retrospective cohort study. The choice of the therapeutic agent (PM or PC), treatment dose, and rate of dose reduction was at the discretion of the attending physicians; therefore, there was a lack of uniformity regarding treatment strategies, which could affect relapse. Second, there is no established definition of RP relapse; thus, we developed our own relapse criteria, using the definition of relapse in a past research on IgG4-related disease [26], namely, (1) worsening of symptoms related to the primary disease and (2) intensified treatment by the attending physicians. This could affect the generalisability of our study findings. Finally, the number of patients was small owing to the rarity of the disease. This may have affected the existence of significant differences. A prospective study with a larger number of patients is required.

## Conclusions

This study analysed factors associated with RP recurrence and showed that tracheal lesions and pre-treatment serum CRP levels were risk factors for recurrence. Initial therapy with prednisolone alone within the first year of treatment was also a risk factor for recurrence. Initial combination therapy with prednisolone and immunosuppressants or biologics may delay recurrence.

## Data Availability

The datasets used and/or analysed during the current study are available from the corresponding author on reasonable request.

## Abbreviations

CI: Confidence interval
CRP: C-reactive protein
ESR: Erythrocyte sedimentation rate
Hb: Haemoglobin
HR: Hazard ratios
IS: Immunosuppressant
PC: Prednisolone combined with immunosuppressants/biologics
Plt: Platelet
PM: Prednisolone monotherapy
PSL: Prednisolone
RP: Relapsing polychondritis
RPDAI: Relapsing polychondritis disease activity index
WBC: White blood cell
